# Correction: Circulating insulin-like growth factor-1: a new clue in the pathogenesis of age-related macular degeneration

**DOI:** 10.18632/aging.101833

**Published:** 2019-02-15

**Authors:** Niccolò Castellino, Antonio Longo, Teresio Avitabile, Andrea Russo, Matteo Fallico, Vincenza Bonfiglio, Mario Damiano Toro, Robert Rejdak, Paolo Murabito, Claudio Furino, Michele Reibaldi

**Affiliations:** 1 Policlinico Gaspare Rodolico-University of Catania, Catania, Italy; 2 Eye Clinic, University of Bari, Bari, Italy; 3 Department of General Ophthalmology, Medical University in Lublin, Lublin, Poland

Original article: Aging (Albany NY) 2018; 10: 4241-4247

**This article has been corrected:** The authors requested to add **Katarzyna Nowomiejska** to the list of authors. **Katarzyna Nowomiejska** was omitted in this publication by mistake. The correct information is provided below. The authors declare that this correction does not change the results or conclusions of this paper. The authors sincerely apologize for this error.

Niccolò Castellino^1^, Antonio Longo^1^, Teresio Avitabile^1^, Andrea Russo^1^, Matteo Fallico^1^, Vincenza Bonfiglio^1^, Mario Damiano Toro^1,3^, Robert Rejdak^3^, Katarzyna Nowomiejska^3^, Paolo Murabito^1^, Claudio Furino^2^, Michele Reibaldi^1^

^1^Policlinico Gaspare Rodolico-University of Catania, Catania, Italy

^2^Eye Clinic, University of Bari, Bari, Italy

^3^3Department of General Ophthalmology, Medical University in Lublin, Lublin, Poland

